# Geological Survey

**Published:** 1847-08

**Authors:** 


### GEOLOGICAL SURVEY.

A corps of geologists has been appointed by government to survey the mineral lands authorized by Congress to be brought into market in the northern part of Wisconsin and the new territory of Minesota. Of this learned body, David Dale Owen, M.D., of New Harmony, is chief; and J. G. Norwood, M.D., of Madison,

B. F. Shumard, M.D., of Louisville, Col. Charles Whittelsey, of Ohio, Prof. Randall, of Cincinnati, and Mr. B. C. Macy, of New York, are his assistants. We look forward to the results of this enterprize of government with great interest. Reports will be made to Congress from time to time, and in the experience and learning of Dr. Owen, and the ability of his co-laborers, the public have a guaranty that the work will be faithfully and skilfully performed. Our young friend, Dr. Shumard, has been toiling assiduously for years past in this interesting department of science, and hitherto without reward or encouragement. He is now in a field where his fine talents will be developed and exhibited, and he has before him a career in which he will win distinction. The service in which he is engaged is expected to continue for several years.

